# Understanding the Participation in Home, School, and Community Activities Reported by Children with Disabilities and Their Parents: A Pilot Study

**DOI:** 10.3390/ijerph16122217

**Published:** 2019-06-24

**Authors:** Ya-Tzu Liao, Ai-Wen Hwang, Hua-Fang Liao, Mats Granlund, Lin-Ju Kang

**Affiliations:** 1 Graduate Institute of Early Intervention, College of Medicine, Chang Gung University, 259 Wen-Hwa 1st Road, Kwei-Shan, Tao-Yuan City 333, Taiwan; cynliao@gmail.com (Y.-T.L.); awhwang@mail.cgu.edu.tw (A.-W.H.); 2 The Department of Physical Medicine and Rehabilitation, Chang Gung Memorial Hospital, Linkou, 5 Fu-Xing St., Kwei-Shan, Tao-Yuan City 333, Taiwan; 3 The School and Graduate Institute of Physical Therapy, College of Medicine, National Taiwan University, 17 Xuzhou Rd., Taipei City 100, Taiwan; hfliao@ntu.edu.tw; 4 CHILD, Swedish Institute of Disability Research, Jönköping University, Gjuterigatan 5, 553 18 Jönköping, Sweden; Mats.Granlund@ju.se

**Keywords:** participation, involvement, school age, children with disabilities, Picture My Participation

## Abstract

Participation has significant impact on children’s health and well-being. Knowledge is limited on how children with disabilities perceive their participation and whether their perceptions differ from their parents. This pilot study aimed to explore whether self-reported frequency of participation and prioritized activities differ between children with disabilities and their parents. Thirty children with disabilities eligible for special education in elementary school and their parents were included. Each of them were interviewed with the Chinese version of Picture My Participation (PMP), separately, to identify the child’s participation frequency in 21 activities at home, school, and community, desire-to-change activities, and the level of involvement in these activities. The results indicated that children’s ratings of participation frequency were significantly lower than parents’ ratings in home activities but not in school and community activities, as analyzed by the Wilcoxon Signed Ranked test. Nineteen (63%) child–parent pairs had selected entirely different items as their desire-to-change activities. Children selected the activities that they were somewhat to very involved in; while parents selected the activities they thought their children were less involved in. Our findings suggest that children with disabilities had unique views on life and this should be supported in their health care and individualized education plans.

## 1. Introduction

Participation has become a significant focus in childhood health care research and practice. In the International Classification of Functioning, Disability, and Health (ICF) [1] and its child and youth version (ICF-CY) [2], participation is defined as the child’s involvement in daily life and is affected by their surrounding environment. Participation in home, school, and community life enables children to develop life skills, social relationships, self-determination, and meaning in life [3,4]. Novel approaches of pediatric health care services focus on optimizing participation through environment support and resources provision based on the child and family priorities [5,6,7]. Assessment of participation in collaboration with the child and family is critical in order to develop effective and feasible intervention strategies.

According to previous research, school-age children with disabilities are more restricted in participation compared with their peers with typical development [8,9,10]. They engaged in less diverse and less intensive leisure activities, were involved in fewer social interactions, and were more subject to environmental restrictions and less activity opportunities [11]. Previous studies are primarily based on parental reports and the child’s voice is rarely heard. The United Nations Convention on the Rights of the Child (UNCRC) asserts the right of every child to express opinions and to participate in decisions that affect their own lives [12]. The Act reinforces children’s rights of self-expression and participation regardless of their age, background, or disability. Research that explores the child’s perspective of participation meets the international policies and has implications for collaborative care planning with the child.

Children and family members both play active roles in the decision making in client-centered care [13]. Parents are usually regarded as the ones who know the best about their children’s needs and are usually the decision makers for children’s health care [13]. However, as children grow older and become more capable of expressing themselves, opportunities should be given to consider the children’s perspectives [14]. Research indicated that conceptions of participation of children with disabilities were related to their age but not the type of disabilities, and they had a different focus from their parents, teachers, and professionals when defining participation [15]. Further, recent evidence has shown that children can actively participate in the goal setting process with support appropriate to their functional level [16]. Children want to be involved in health-related discussions but have not been given appropriate opportunities [17]. Allowing children to express and make decisions for themselves might lead to higher learning motivation and self-determination [18]. Children with communication difficulties can be actively involved in expressing opinions if methods such as pictures support or alternatives to manipulate response are provided [19,20]. Such adaptations need to be utilized for the assessment of participation.

Systematic reviews suggest that participation involves attendance and involvement as two essential constructs [21,22]. Attendance is defined as “being there” in activities and can be assessed by measuring the frequency of attending, the duration of attending, and the diversity of activities attended. Involvement is defined as the experiences while “being there” in activities which might include engagement, motivation, persistence, social relationship, and affects [22]. Measures of participation involving attendance and involvement can provide a picture of the actual daily life performance of children, and may facilitate the children’s expression of their own wishes for participation based on an individual child’s strengths and life experiences in their natural contexts. The outcomes of assessing participation may expand knowledge on the different views on life of child and proxy-respondents [20,23,24,25]. A recent review of participation measures shows that few measures provide information on both attendance and involvement and even fewer are designed as self-rating measures where children with disabilities can self-rate their perceptions of participation [26].

In an ongoing project, a new self-rating instrument, Picture My Participation (PMP), is designed to capture self-perceived participation of children with disabilities in everyday activities [27]. The development of PMP was based on the ICF and UNCRC in collaboration with United Nations Children’s Fund (UNICEF) [27]. The items were derived from reviewing existing measures of participation and matching items to the UNCRC and Conventions of the Rights of Persons with Disabilities (CRPD) [28]. The items were selected for children with disabilities, particularly those from marginalized communities and low socio-economic environments, by consulting with these children and communities, as well as representative of the Activities and Participation chapters of the ICF-CY [2]. The PMP includes 20 items focusing more on the home (9 items) and community (10 items) domains with less focus on school domain (1 item). The 20 activity items appear to cover essential aspects of everyday activities for children with intellectual disability in a low and middle income setting (e.g., South Africa) as well as a high-income setting (e.g., Sweden) [28]. A specific aim of PMP is to allow children to express their own opinion. As a means to this end, it is important to compare perceptions of participation of children and parents responding to the same questions already on the validation phase of instrument development. Probably children who can express their own opinion, and thus influence intervention decisions, are more motivated to engage in participation interventions [18]. 

This pilot study aims to explore the children’s and parents’ reported frequency of participation in home, school, and community activities, their desire-to-change activities, and the level of involvement in these desired activities with the help of the PMP instrument. 

## 2. Materials and Methods

### 2.1. Participants

The children with disabilities and their parents were recruited using a convenient sampling method through researchers’ networking. The children were between the ages of 6 and 13 years, formally receiving special education services granted by the Special Education Students Diagnosis and Placement Counseling Committee in Taiwan, and enrolled in grades 1 to 6 at public elementary schools. To be included, the children needed to have basic communication and interaction skills that allowed them to understand the content of the instrument, to make choices between 4 options, and to express their thoughts verbally or with Augmentative and Alternative Communication (AAC). Children with uncorrected visual or hearing impairments, and who were therefore unable to see or hear during the interview, were not included. The parents needed to understand and speak Mandarin Chinese. Ethical Institutional Review Board approval was obtained from the Chang Gung Memorial Hospital in Taiwan, and all the children and parents provided the informed consents.

A total of 30 children (20 boys and 10 girls) and 28 parents (23 mothers, 4 fathers, and 1 grandmother) participated in this study. Two of the mothers each had two children participating in the study. Among the 30 children, 27 attended decentralized resource classes, meaning they were included in the mainstream classrooms for most of school hours. Children were diagnosed with various conditions, with Autism Spectrum Disorder being the most prevalent (33%) (Table 1). Three children with Autism Spectrum Disorder had limited verbal skills and used AAC to facilitate communication; among them, one had a disability certificate with a severe degree of disability. The other children could communicate verbally and had mild to moderate degrees of disability. Though their verbal communication abilities varied, all children could follow instructions and respond to the questions verbally or by pointing. The domicile of origin show 25 parents from Taiwan, 2 from Vietnam, and 1 from China, all of them were able to communicate in Mandarin Chinese (Table 2).

### 2.2. Instrument

#### 2.2.1. Picture My Participation

Picture My Participation (PMP) is a newly developed self-reporting instrument for a child to talk about his/her participation in 3 domains: home, school, and community [27]. An interview approach called Talking Mats™ [29] was used in which the PMP items and response options were converted into graphic symbols or visual scales to facilitate conversation with the child. Talking Mat™ [29] is an easy-to-use visual method using a mat with picture symbols attached, which is an efficient tool to support people with communication difficulties to be actively involved in research [30]. Picture symbols used to present each PMP activity were selected from the Picture Communication Symbols (PCS™) as part of the Boardmaker™ software program by DynaVox Mayer-Johnson, LLC (Pittsburgh, PA, USA) [31]. The Boardmaker™ is commercially available and has been adapted to different languages and used worldwide. Three separate mats were displayed for the child to identify: (1) Frequency of participation in home, school, and community activities; (2) three prioritized activities that the child desires to change; and (3) perceived level of involvement in these selected activities. On the ‘frequency’ mat, the visual scale presented a six-point Likert scale: 6 (Always, the child participates all of the time), 5 (Sometimes, the child participates some of the time), 4 (Not really, the child occasionally/rarely participates), 3 (Never, the child does not participate), 2 (Not applicable), and 1 (Unsure or no answer, the child does not know the answer or does not answer at all). The “desire-to-change” mat allowed the child to place the PCS™ symbols indicating their prioritized activities. Prioritization of desire-to-change activities is based on the child’s wishes to do an activity more often or less often, or to perform an activity which they do not have an opportunity to. On the “level of involvement” mat, the visual scale presented a five-point Likert scale: 5 (Very involved), 4 (Somewhat involved), 3 (Minimally involved), 2 (Not applicable), 1 (Unsure or no answer). Level of involvement was further explained as the extent to which the child enjoyed or focused on the activities.

Content validity of the PMP has been established on a sample of 149 children with and without intellectual disabilities in South Africa (*n* = 112) and Sweden (*n* = 37). All items were selected as important by children with intellectual disability in both countries, representing low- and middle-income, and high-income settings. All levels on the scales have been used to rate the frequency of participation and level of involvement for each activity. The relevance of content in terms of selection of items and use of scales appeared to be higher for children with intellectual disability than for children with typical development [29]. 

#### 2.2.2. Translation of the PMP-Chinese Version

The PMP has been translated into a Chinese version (PMP-C) and then re-translated into English followed a standardized cultural adaption process of self-report measure [32]. A translator with a professional background (in pediatric rehabilitation) and another with a non-professional background (an English teacher) carried out the forward translation, respectively. Both translated versions were synthesized to form a Chinese translation version. Then, a person who had never seen the instrument, and whose native language is English, performed the backward translation, and the translated contents were presented to the group of original authors to ensure the translated items accurately reflected the meaning of the original items. At the time of this study the same sets of PCS™ symbols in the original PMP were used. A pilot testing was conducted by interviewing 7 elementary school students with disabilities to explore their thoughts about the feasibility of the PMP in capturing their prioritized activities in life. Although the validity and suitability of the picture symbols were not formally tested, all children in the pilot testing were able to understand the picture symbols with the interviewer’s explanation during the interview. All children were able to identify up to 3 prioritized activities; and 5 out of 7 students expressed different opinions toward the item “Formal learning at school.” They desired activities such as going out or playing with classmates and joining after-school clubs that were not in the original PMP items. As a result, one additional item, namely, “Play and leisure at school” was considered important and thus was added for the school domain, resulting in 21 items in the PMP-C used in this study [33]. The translation and adding of the items were approved by the authors. Our recent study further provided cultural validity of this translated version [34]. Semi-structured cognitive interviews were conducted with five children 6–13 years old with special needs and typical development, and their parents to examine the conceptual, item, semantic, and operational equivalence based on the Applied Cultural Equivalence Framework [35]. Children and parents considered this instrument appropriate to measure children’s expression of thoughts and life experiences in Taiwan. Though suggestions on the items and operational procedures were provided, children and parents were able to understand the concepts measured in this instrument and the meanings of items, indicating initial evidence of cultural validity [34].

### 2.3. Study Design and Procedures

The PMP-C was used in an interview by a trained researcher with the children and parents, separately, to enable a comparison of the child’s own perspectives and parents’ proxy information. For the children, a semi-structured interview was conducted to guide the children to respond to the PMP-C with the Talking Mats™ procedure as described above. There were three steps to conduct the interview. First, the researcher showed the “frequency” mat and explained what each frequency means to the child, then trialed the first activity to see if the child understood how to rate the activity. The researcher explained every activity and the child placed each activity symbol on the visual scale of frequency to indicate their responses until finishing the 21 activities. Second, the researcher showed and explained the “desire-to-change” mat. The child was asked to select three activities that he/she wishes to make changes to that have been displayed in the “frequency” mat. We allowed flexibility for the child to select more or less than 3 activities if they wanted to. Third, the researcher showed and explained the “level of involvement” mat and then the child rated “level of involvement” by placing the selected activities on the visual scale of level of involvement. Parents first completed the demographic questionnaire, then the researcher interviewed the parents to complete the PMP-C. The demographic questionnaire was used to obtain basic information including child sex, age, medical conditions, type of special education, living area, primary caregivers, family type, and socioeconomic status. In the PMP-C interview, parents provided ratings for their children’s participation frequency, selected up to three “desire-to-change” activities for their children, and rated the level of involvement of these selected activities for their children. Children and parents were always asked to provide reasons for their rating and selection for each activity, to share their own stories and thoughts behind the selection.

### 2.4. Statistical Analysis

Statistics analysis was conducted by IBM SPSS Statistics 21.0 (Armonk, NY, USA). Children and parents’ frequency scores for the PMP-C were not normally distributed, thus a non-parametric analysis Wilcoxon Signed Ranked test was used to compare participation frequency reported by the children and parents. Non-applicable and no-answer items, if any, were not included in the analysis of this study. Matched desire-to-change activities between children and parents were examined by calculating the number and percentage of the same activity selected by each parent and child dyad. The frequency counts of each activity being selected as “desire to change” was calculated, and the top 5 activities were listed to present the difference in the distribution of choices between children and parents. Descriptive statistics (mean and SD) were calculated to present level of involvement across the activities selected by children and parents.

## 3. Results

### 3.1. Comparison of Frequency of Participation

All 30 children and 28 parents were able to complete the PMP-C interviews. Table 3 presents the results for comparing the frequency scores between children and parents. Children’s frequency scores were significantly lower than parents’ scores in total and home domain scores. Children perceived doing home activities less often than did their parents. The descriptive statistics also showed that children perceived participating less often in community activities; while at school, children perceived participating more often than did their parents. The results for community and school domains, however, did not reach significance.

### 3.2. Comparison of Desire-to-Change Activities 

All 30 children were able to select 1–4 desire-to-change activities (total 91 activities), and all parents selected 1–3 desire-to-change activities (total 80 activities) for their children based on the PMP-C items (Table 4). Among the identified desire-to-change activities, most of the child’s priorities were related to community domain (44%); while most of the parents’ priorities were related to home domain (46%). To further analyze the matched activities selected by every parent-child dyad, we found that 19 dyads (63%) have selected totally different desire-to-change activities, 10 dyads (33%) selected 1 same activity, and only 1 dyad (3%) selected 2 same activities. Detail list of selected activities by the children and parents is shown in Appendix A.

**Figure 1:**
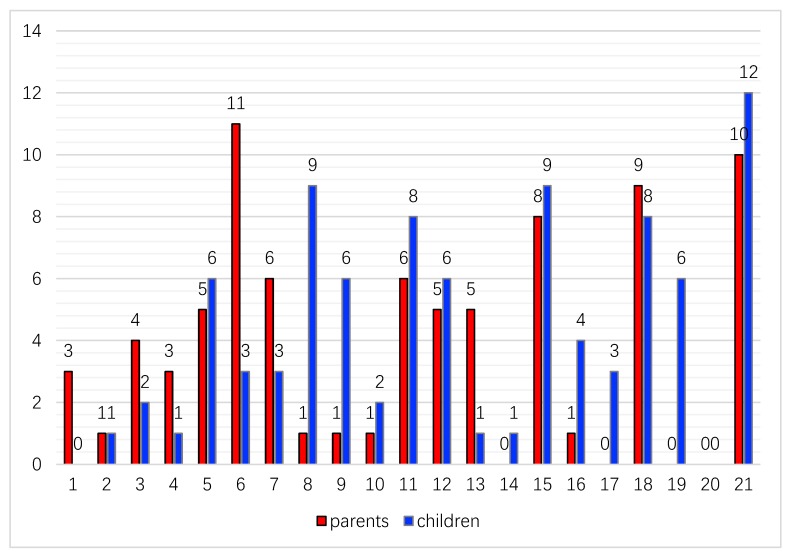
Frequency count of each item of the PMP-C being selected as desire-to-change activity by children and parents. Item names: 1st. Personal care; 2nd. Family mealtime; 3rd. Looking after one’s own health; 4th. Gathering daily necessities for family; 5th. Meal preparation with or for the family; 6th. Cleaning up at home; 7th. Taking care of other family members; 8th. Taking care of animals; 9th. Interact with the family; 10th. Family/community celebrations; 11th. Getting together with other children in the community; 12th. Organized leisure; 13th. Quiet leisure; 14th. Religious and spiritual activities; 15th. Shopping and errands; 16th. Social activities in the community; 17th. Visit to health center; 18th. Formal learning at school; 19th. Overnight visits and trips; 20th. Paid and unpaid employment; 21th. Play and leisure at school. Note: PMP-C = Chinese version of Picture My Participation.

The frequency count of each activity being selected as a desire-to-change activity was presented (Figure 1). Children identified desires for change across 19 items; while parents identified desires for change across 17 items. Item 20 “paid and unpaid employment” was not relevant to both the children and their parents in this study. Children’s top five selections were: Item 21 (Play and leisure at school, 40% of the children selected this item), item 15 (Shopping and errands, 30%), item 8 (Taking care of animals, 30%), item 11 (Getting together with other children in the community, 27%), item 18 (Formal learning at school, 27%). Parent’s top five selections were: Item 6 (Cleaning up at home, 37% of the parents selected this item), item 21 (Play and leisure at school, 33%), item 18 (Formal learning at school, 30%), item 15 (Shopping and errands, 27%), item 11 (Getting together with other children in the community, 20%), and item 7 (Taking care of other family members, 20%). Items 11 and 7 had the same frequency counts so they were tied for fifth place of the parents’ top selections.

Item 15 (Shopping and errands), item 18 (Formal learning at school), and item 21 (Play and leisure at school) are common activities among top selections for both children and parents. On the other hand, some activities showed distinct results between parents and children. For example, item 6 (Cleaning up at home) is on top of parents’ lists; however, it is selected by 3 children only. Item 8 (Taking care of animals) is selected by 9 children but only 1 parent, and item 19 (Overnight visits and trips) is selected by 6 children but not any parent. 

### 3.3. Level of Involvement for Desire-to-Change Activities

**Figure 2:**
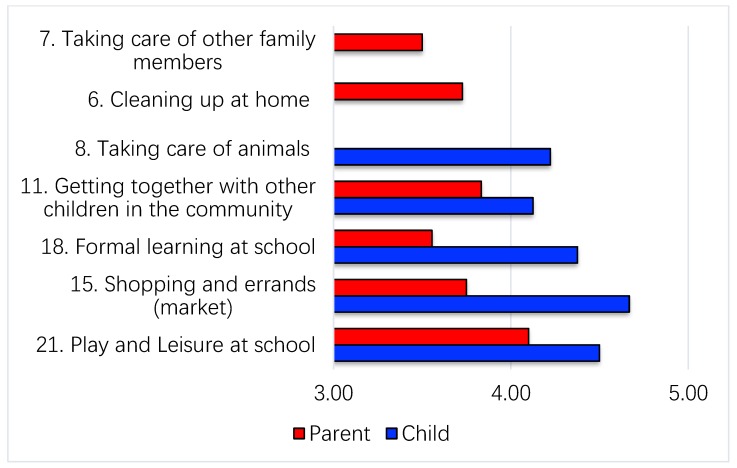
Mean scores for level of involvement of top 5 or 6 activities of the PMP-C selected and rated by children and parents. Level of involvement scoring: 5: Very involved, 4: Somewhat involved, 3: Minimally involved, 2: Not applicable, 1: Unsure or no answer. Note: PMP-C = Chinese version of Picture My Participation.

Children and parents rated the level of involvement for each selected desire-to-change activity. The mean scores for level of involvement of 91 activities selected by the children was 4.33 (in between somewhat involved to very involved, SD = 0.78), while the mean scores of 80 activities selected by the parents was 3.71 (in between minimally involved to somewhat involved, SD = 0.69), indicating that children generally rated themselves as more involved in their selected activities. The mean scores for level of involvement of the children’s top five selected activities all fall in between somewhat involved to very involved; while the mean scores of the parents’ top six selected activities all fall in between minimally involved to somewhat involved (Figure 2).

## 4. Discussion

The PMP-C is an innovative instrument that helps to advance participation-based practices for children with disabilities. The results of the PMP-C indicated that the children in general perceived doing activities less often than what their parents perceived, especially in home activities. The result signifies the importance of actually asking the children themselves about their perceived participation. Previous studies have also shown that children and their parents had different views on participation and quality of life for children with various health conditions in different cultures [24,36,37,38]. For example, adolescents with cerebral palsy in Turkey reported higher levels of daily functioning in upper-extremity activities, sports and physical activities, and happiness than their parents [24,36,37,38]. Children with ASD in Iceland reported a higher level of quality of life than did their parents [38]. Children with disabilities in Taiwan, as shown in this study, reported lower levels of participation in various home-related activities. The items under home domain of the PMP-C include self-care, chores, and social-related items. Interestingly, when looking at each individual item, the children in general reported a lower frequency of participation than did their parents in social-related items, such as interact with the family, family mealtime, meal preparation with or for the family, taking care of other family members; while children reported a higher frequency than did parents on self-care and chores-related items, such as daily routines for personal care, cleaning up at home, and taking care of animals or pets. Common reasons provided by the children were that their parents came home late due to work, or they were busy doing homework, and therefore did not spend enough time interacting with their families. The results meaningfully reflect the children’s perspectives about limited family participation given long school- and working-time in our society.

Children and their parents identified different priorities for participation. A majority of the child–parent pairs in this study had no correspondence in selecting the activities they wished to change based on their life experiences. Emerging evidence also suggests that children with disabilities and their caregivers value different things. For example, a study has suggested the incongruence of children’s and their caregivers’ goals for school-based therapy services [25]. Children wanted to perform better in various life skills including self-care, school, and leisure, and parents focused on school task performance, while teachers tended to set goals related to motor and cognitive skills [25]. Even when the child and parent selected the same activity, they might have different rationale. As indicated in Appendix A, a particular example is “Formal learning at school”. The parents usually select this activity as they want their children to do better on academic performance. The children, on the other hand, reported that they wish to have less homework and study, so they can spend more time with classmates or friends after school. Therefore, it is important to ask follow-up questions after the child selects an activity to probe into the real desires behand the selection. 

The mean values for level of involvement indicated that children selected desire-to-change activities in which they were more involved; while parents selected activities they thought their children were less involved in. As we learned from the interviews, children tended to prioritize activities that they liked to do, and were willing to do more of. Parents, on the other hand, tended to prioritize activities they expected the child to improve or be more involved in. The results again supported that children and parents have different expectation for their lives and this should be respected [19,24]. Interviewing with the PMP-C offers opportunities to hear children’s voices that inform decision making in collaboration with parents and professionals.

The PMP-C provides a useful tool in supporting children with disabilities to express views that are important for goal-setting. With structural guidance and visual cues, the children completed their activity cards sorting on the talking mats; in the meantime, they also further explained their own experiences and expectations. The complete array of cards helps children to select activities at a glance. These approaches such as semi-structured interviews or self-report scale with picture tools are frequently found in health care research involving children and youth with disabilities [19]. In addition, the PMP-C interview session for each child took around 40 minutes which is suitable for the attention span of a school-age child. 

With the appropriate support, all the children in this study were able to successfully selected desire-to-change activities. Depending on the child’s different needs for understanding and expression, the researcher used different verbal or visual guidance to facilitate communication during the interviews. In particular, most children in this study did not have experiences in expressing goals and wishes or making a decision; which made it hard to determine “desire-to-change” activities. In such case, they were encouraged to consider those activities they less often participated in and, thus, wished to participate more often in. Conducting a child interview requires coaching skills and coping strategies that are appropriate for their functional level and past experiences [19]. 

The PMP-C has potential implications for involving children with disabilities in making decisions for their own care. Decision-making is a continuous process that requires intervention to facilitate children’s expression, such as age or ability-appropriate techniques, and creative visual methods [17]. The positive attitudes and communication skills of professionals play a significant role in supporting children’s self-expression and decision-making [39]. That, together with the use of an appropriate tool, would further facilitate children to express participation experiences and desire to change activities. The PMP-C provides ratings that help quantify frequency of participation and level of involvement perceived by the children and parents. An additional value is that children were encouraged to elaborate on their feelings and experiences when sorting the activity cards. The interview is a process to interact and communicate with the child, not only to obtain “scores” from the child. Our study protocols demonstrated a means to effectively share responsibility and engage children in decision making in ways that are functionally appropriate and acceptable to families.

This study has several limitations and is thus in need of future study. The participants involved was a convenience sample of a limited number of children whose age distribution was mostly in middle and upper grades of elementary school; thus was not a representative sample of school-age children with disabilities in Taiwan. Children’s functional abilities, such as cognition, motor, or communication, were not measured using standardized assessment, hence detailed information of a child’s functioning was lacking for further analysis. The PMP-C is still under development and further research is needed to enhance cultural validity for the use with Taiwanese children. To elaborate, the picture symbols in the original PMP were used in this study and children were sometimes confused by the pictures that were not consistent with their experiences in Taiwan. For example, a picture of rainbow for item 14 “Religious and spiritual gatherings and activities” may be vague to the children. An effort to design pictures specific to Taiwanese culture would further improve the relevance of the PMP-C items in Taiwan [34]. This study supported the feasibility as part of the instrument development by demonstrating that children with disabilities were able to identify their experiences and goals through the picture-supported interviews of PMP-C. Further studies are needed to develop strategies that engage children in goal-setting jointly with parents and professionals, to obtain and integrate support from others such as teachers and peers, and to measure the achievement of participation-based goals in children’s health care and individualized educational plans.

## 5. Conclusions

School-age children with disabilities have different views and wishes of participation when compared to their parents. Children with disabilities and their parents identified different activities they desired to change in life through the use of the PMP-C. Children tended to select the activities they liked to do, and were willing to do more of; while parents tended to select the activities they want the child to do better in. Further works are needed to provide a culturally relevant instrument and procedures that actively involve children with disabilities in collaborative goal-setting in health care and individualized educational plans.

## Figures and Tables

**Table 1:** Demographic information of children (*n* = 30).

Variables	*n*	%
Child’s age		
8 years	2	7
9 years	5	17
10 years	9	30
11 years	6	20
12 years	8	27
Having a disability certificate		
Yes, with a mild degree of severity	13	43
Yes, with a moderate degree of severity	4	13
Yes, with a severe degree of severity	1	3
No ^a^	12	40
Educational placement		
Decentralized resource classes	27	90
Centralized special class	3	10
Health conditions ^b^		
Autism Spectrum Disorder	14	47
Learning disability	8	27
Developmental delay	6	20
Emotional disability	6	20
Intellectual disability	5	17
Attention Deficit Hyperactivity Disorder	5	17
Speech & Language Impairment	2	7

^a^ Children with a medical diagnosis and were eligible for special education, but have not been issued a disability certificate. ^b^ A child may have more than one condition as reported by parents.

**Table 2:** Demographic information of parent respondents (*n* = 28).

Variables	*n*	%
Relationship to the child		
mothers	23	82
fathers	4	14
grandmother	1	4
Age (years)		
30–39	10	36
40–49	14	50
>50	4	14
Nationality (domicile of origin)		
Taiwan	24	86
Vietnam	3	11
China	1	4
Educational background		
Illiterate	1	4
Elementary school	2	7
Junior	3	11
High school	7	25
Junior college	4	14
University	11	39
Family annual income ^c^		
<300k	12	43
300k to 2M	16	57

k = thousand; M = million. ^c^ The average family annual income in Taipei City is New Taiwan Dollars 1,568,945. (NTD$30 = USD$1) According to the Taipei City Government, if the average household income divided by the total household population is less than NTD 16,157, each person will receive a low-income living allowance per month. The study was based on a minimum of 2 people per household, and total income below 300k is considered as a low-income household.

**Table 3:** Comparison of the PMP-C frequency of participation between children and parents’ ratings.

PMP-C	Child			Parent			*p* Value
	25th	Median	75th	25th	Median	75th	
Total	4.49	4.65	4.86	4.55	4.80	5.01	0.040 *
Home	4.44	4.67	5.11	4.73	5.00	5.34	0.018 *
School	5.00	5.50	6.00	5.00	5.25	5.5	0.079
Community	4.22	4.44	4.69	4.22	4.50	5.00	0.114

Frequency scoring: 6: Always, 5: Sometimes, 4: Not really, 3: Never, 2: Not applicable, 1: Unsure or no answer. Note: PMP-C = Chinese version of Picture My Participation. * *p* < 0.05.

**Table 4:** Comparison of the desire-to-change activities of children selected by the children (*n* = 30) and parents (*n* = 28) ^a^.

Number of Activities	Child	Parent
Total	91 (100%)	80 (100%)
Home domain	31 (34%)	37 (46%)
School domain	20 (22%)	19 (24%)
Community domain	40 (44%)	24 (30%)

^a^ Two of the parents each selected for their 2 children.

## Abbreviations

The following abbreviations are used in this manuscript:

ICF	International Classification of Functioning, Health, and Disability
UNCRC	United Nations Convention on the Rights of the Child
PMP-C	Chinese version of Picture My Participation
UNICEF	United Nations Children’s Fund
AAC	Augmentative and Alternative Communication

## Appendix A

**Table A1:** Item numbers of the PMP-C selected as a desire-to-change activity of all children and their parents.

Child No.	Child				Parent			# of Same Selection
	1st	2nd	3rd	4th	1st	2nd	3rd	
1	8th	11th	16th		6th	15th	18th△	0
2	4th	12th	15th		15th	16th	18th	1
3	9th	19th	21th		3rd	15th	21th	1
4	6th	9th	18th—		13th	18th		1
5	12th	8th	11th		6th	18th		0
6	19th	9th	15th		18th△	12th△	1st	0
7	11th	15th	18th	19th	6th	7th	5th	0
8	7th	8th	17th		3rd	7th	21th	1
9	11th	18th	21th		11th	13th	21th△	2
10	3rd—	5th	8th	17th	5th	11th	21th	1
11	8th	19th			13th			0
12	19th	8th	18th—		5th	6th	7th	0
13	5th	6th	19th	7th	18th△	21th		0
14	5th	12th	16th		1st △	5th	18th△	1
15	12th	11th	21th		7th	15th	4th	0
16	18th—	15th	12th		3rd	15th	21th	1
17	18th	9th	5th		12th	21th		0
18	18th—	13th			11th	18th△	21th	1
19	8th	21th	15th		6th	13th	7th	0
20	8th	15th	21th	12th	6th	13th	7th	0
21	21th	16th	5th		3th	6th	4th	0
22	18th —	21th	17th—		6th	8th	1st	0
23	15th	11th	10th		21th	15th		1
24	9th	21th			10th	11th	12th	0
25	21th	2nd			5th	6th	11th	0
26	21th	7th	8th		6th	15th	18th△	0
27	21th	11th	9th		4th	6th	12th	0
28	15th	14th	3rd	21th	15th	11th		1
29	11th	5th	6th		12th	2nd	21th	0
30	10th	16th	15th		9th			0

Item with gray background highlights same selection between the child and parent. Items being selected with an intention to do less are marked with “—”; Items being selected with an intention to be more involved or be independent are marked with “△”; other unmarked items indicate an intention to do more. Item names: 1st. Personal care; 2nd. Family mealtime; 3rd. Looking after one’s own health; 4th. Gathering daily necessities for family; 5th. Meal preparation with or for the family; 6th. Cleaning up at home; 7th. Taking care of other family members; 8th. Taking care of animals; 9th. Interact with the family; 10th. Family/community celebrations; 11th. Getting together with other children in the community; 12th. Organized leisure; 13th. Quiet leisure; 14th. Religious and spiritual activities; 15th. Shopping and errands; 16th. Social activities in the community; 17th. Visit to health center; 18th. Formal learning at school; 19th. Overnight visits and trips; 20th. Paid and unpaid employment; 21th. Play and leisure at school. Note: PMP-C = Chinese version of Picture My Participation.
